# Infamous Gaming: The Intergroup Bias of Non-gamers in the Chinese Marriage Market

**DOI:** 10.3389/fpsyg.2022.682372

**Published:** 2022-02-07

**Authors:** Shuguang Zhao, Wenjian Zhang

**Affiliations:** ^1^School of Journalism and Communication, Renmin University of China, Beijing, China; ^2^Institute of Zijin Media Research, Nanjing University, Nanjing, China

**Keywords:** attitude toward gaming, mate selection, gamers, non-gamers, intergroup bias

## Abstract

The link between gaming and negative outcomes has been explored by previous research and has led to the widespread adverse attitude toward gaming (ATG) and gamers, especially from those who are unfamiliar with this activity. By implementing an audit study with gamers and non-gamers as participants (*N* = 1,280), we found that non-gamer participants rated gamers less as similar to their ideal marriage partners compared to non-gamers, while gamer participants did not differentiate between gamers and non-gamers in the ideal marriage partners similarity rating (IMPSR). The findings also revealed that the difference in IMPSR between gamer and non-gamer participants toward gamers was completely mediated by their ATG. These results imply that non-gamers consider gaming as an undesired characteristic, and this is due to the relatively negative attitude of non-gamers toward gaming. Current study provides a new perspective on exploring the effect of gaming by investigating the social interaction between gamers and non-gamers in real-world and suggests that the unfamiliarity of gaming can lead to the negative ATG, which may, ultimately, place gamers at a disadvantage in the context of mate selection.

## Introduction

Video games have become an increasingly popular form of entertainment (Williams et al., 2008) over the past decades. Most existing game-related research has focused on the impact of gaming (Anderson and Bushman, 2001), and on the interaction between gaming and gamers (Caroux et al., 2015), while insufficient attention has been paid to the social interaction between gamers and non-gamers. In fact, there were 484 million gamers in China accounting for 58.4% of Chinese Internet users as of 2019 (CNNIC, 2019). Gamers—the label used to refer people who play games—have become a huge social category (Nauroth et al., 2014). However, gaming has been widely related to diverse negative outcomes, such as aggressive behavior (Gentile et al., 2004; Carnagey et al., 2007; Zhang et al., 2021) and psychological dysfunction (Gentile et al., 2011; Tortolero et al., 2014). Nevertheless, other studies have suggested that gaming may not be a source of real-world negative outcomes (Ferguson, 2010; Valadez and Ferguson, 2012; Király et al., 2017; Ferguson and Wang, 2020), highlighting that there is little consensus on the relationship between negative outcomes and gaming.

According to the moral panic theory (Cohen, 1972), the negative effects of innovation may be exaggerated because of misunderstanding, which could explain the perceived link between negative real-world outcomes and video games (Ferguson, 2008, 2018; Ferguson and Colwell, 2017). This belief has gained widespread popularity, with a poll conducted in 2013 showing that the majority of Americans believed that video games were strongly associated with violent behavior (Harris Interactive, 2013). This idea is even more evident among those who lack concrete gaming experience (Przybylski, 2014). Simply stating “playing games is bad and not playing games is good” may promote moral superiority and the perceived threat of non-gamers toward gamers, and eventually foster discrimination against gamers.

If discrimination against gamers does exist, it may lead to antagonism between these and non-gamers, as well as to the unfair treatment of gamers in different situations. In addition, prejudice against gamers can be a cover for other forms of discrimination (Markey et al., 2020). For this reason, the current study aimed to examine whether this discrimination extends to the context of mate selection, a field that can fully reflect people’s value orientation. As a relatively new form of entertainment, misgivings toward video games may be inevitable because of unfamiliarity (Bowman, 2016). In contrast, concrete gaming experience has a significant impact on attitudes toward gaming (ATG). Ferguson et al. (2017) found that older adult participants’ negative ATG decreased significantly after playing video games (Ferguson et al., 2017). Another empirical study showed that participants with concrete video game experience were less likely to associate video games with aggressive behaviors (Przybylski, 2014), which may be due to their familiarity with gaming (Ivory and Kalyanaraman, 2009). In this study, we predicted that the moral panic of non-gamers toward gaming may be projected into gamers. Additionally, compared to non-gamers, gamers tend to feel more positive toward gaming as it might help them maintaining a positive social identity (Nauroth et al., 2014). Consistent with this viewpoint, previous studies have showed that gaming experiences lead to a more positive ATG (Przybylski, 2014; Ferguson et al., 2017). For these reasons, we make two hypotheses as follows:

*Hypothesis 1*: Non-gamers would rate gamers as less like their ideal marriage partners, and this rating would be moderated by whether the potential partner was a gamer or not.

*Hypothesis 2*: Compared to non-gamers, gamers would not consider fellow gamers to be less appealing marriage partners, and this would be due to their positive ATG. In other words, this difference in rating between gamers and non-gamers would be mediated by their ATG.

## Materials and Methods

Audit studies have been widely applied in studying discrimination (Neumark, 1996; Milkman et al., 2012; Gaddis, 2015). We implemented an audit study on *Qualtrics*^1^ by distributing the anonymous link on social media, and a total of 1,280 participants (63.2% male), with ages ranging from 18 to 60 years (mean age ± SD = 29.99 ± 7.09 years), were recruited in China. The experiment was approved by the Ethics Committee of Naing University. All subjects filled out the questionnaires online *via* an anonymous link. We collected demographic information, such as age and gender, in the first part of the questionnaire. After that, participants were asked to give an ideal marriage partners similarity rating (IMPSR) toward a fictional marriage partner, after reading the description [“Based on the characteristics described above, how similar do you think he/she is to your ideal marriage partner? (1 = not similar at all, 9 = very similar)”]. Each participant read one of a pair of descriptions, depending on the randomly assigned condition (gamer condition or non-non-gamer condition). The descriptions under these two conditions were identical in every respect—physical attractiveness, level of education, and socioeconomic status—except for whether he/she was a gamer or not (for the gamer condition: “his/her hobby is gaming”; for the non-gamer condition: “he/she has no gaming experience”). After the rating, to ensure participants had assimilated this critical information, they were asked to recall whether the fictional marriage partner described in the previous section was a gamer (“Does the character described in the text have the experience of playing video games?”), the data of subjects with wrong answers were excluded from the data analysis. The participants were then required to report whether they were gamers themselves (“Have you played video games in the last 6 months?”), and their ATG [“Generally speaking, what is your attitude toward gaming”? (1 = strongly oppose, 9 = strongly support)”]. Before completing the questionnaire, all participants provided written informed consent. Considering that gender is an important variable in marriage-related research, we also add gender as predictor in our data analysis.

## Results

All data in the present study were analyzed using SPSS 25.0. A three-factor ANOVA with a 2 (participant type) × 2 (target type) × 2 (gender)was implemented to test Hypothesis 1. The moderating effect of participant type gender was examined as *post-hoc*. Besides, in order to test our Hypothesis 2, a mediating model was also estimated by using ATG as mediator, participant type as independent variable, and IMPSR as dependent variable. Moderating and mediating models were estimated by using the PROCESS v3.5 computation tool (Hayes, 2013). Using the 5,000 samples bootstrapping procedure, 95% bias-corrected confidence intervals (CIs) were generated, and the mediating effects were significant if zero was not contained within CIs.

**Figure 1:**
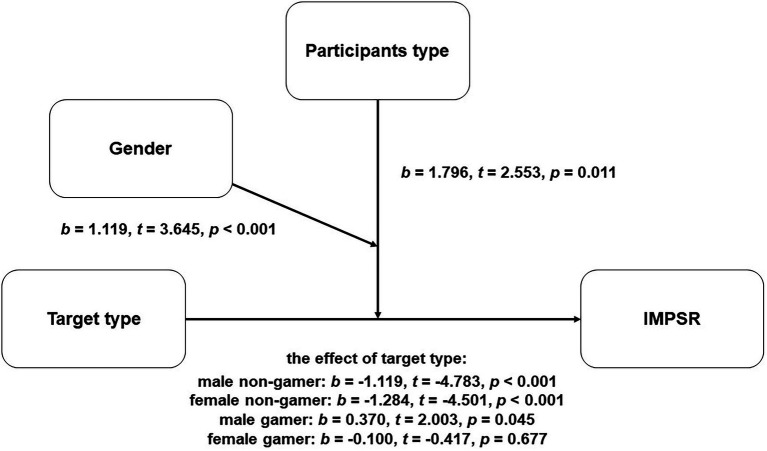
The moderating effect model of participant type (gamer or non-gamer). IMPSR, ideal marriage partners similarity rating.

As a result, 622 and 658 participants were randomly assigned to the gamer and non-gamer conditions, respectively. According to the participants’ subjective reports, 702 and 578 participants were gamers and non-gamers, respectively. As the result of the ANOVA, the main effect of participants types (*F* = 10.048, *p* = 0.002), target types (*F* = 19.968, *p* < 0.001) and the interaction between participant types and target types (*F* = 31.373, *p* < 0.001), and the interaction between participant types and gender (*F* = 16.380, *p* < 0.001) was significant, while the other two interaction effects and the main effect of gender were not significant [gender: (*F* = 2.094, *p* = 0.148); target types and gender interaction: (*F* = 1.769, *p* = 0.184); and three ways interaction: (*F* = 0.411, *p* = 0.522)] being a gamer moderated the impact of gaming on IMPSR. To investigate whether being a gamer moderated the negative impact of gaming on IMPSR, a corresponding moderating effect model was estimated by coding non-gamer as “0” and gamer as “1.” To be specific, among the non-gamer participants, both male and female perceive non-gamer targets as more ideal marriage partner {the effect of target type: male non-gamer: [*b* = −1.119, *t* = −4.783, *p* < 0.001, 95% CI = (−1.578, −0.660)]; female non-gamer: [*b* = −1.284, *t* = −4.501, *p* < 0.001, 95% CI = (−1.843, −0.724)]}. However, male gamers prefer to make intimate relationship with gamers than non-gamers while female gamers show no significant preference {the effect of target type: male gamer: [*b* = 0.370, *t* = 2.003, *p* = 0.045, 95% CI = (0.008, 0.733)]; female gamer: [*b* = −0.100, *t* = −0.417, *p* = 0.677, 95% CI = (−0.570, 0.370)]; see Figure 1}.

**Figure 2:**
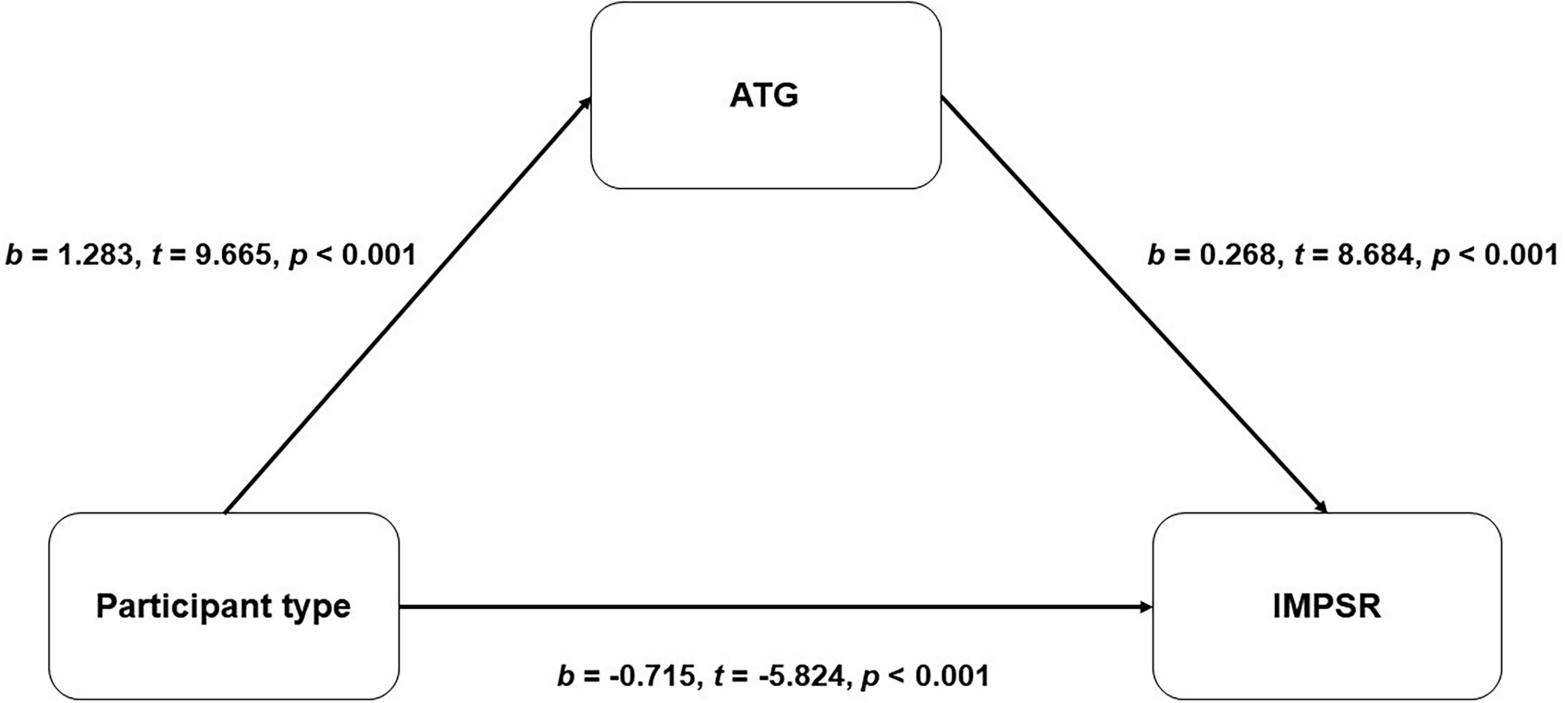
The mediation effect of ATG between participant types and IMPSR for garners among male participants. ATG, attitude toward gaming; IMPSR, ideal marriage partners similarity rating.

**Figure 3:**
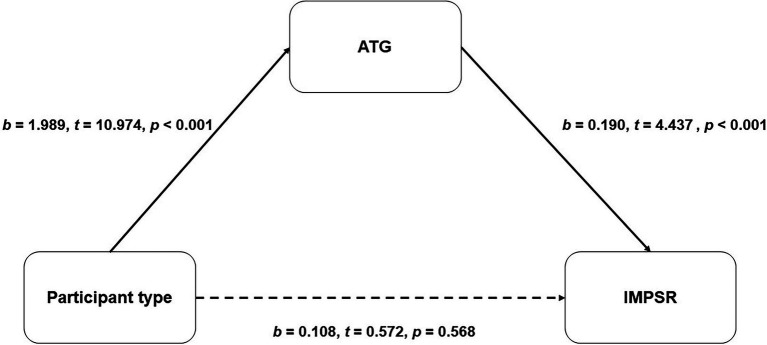
The mediation effect of ATG between participant types and IMPSR for garners among female participants. IMPSR, ideal marriage partners similarity rating.

The coefficients and statistics of the mediating effect models are presented in Figures 2, 3. Among male participants, a partial mediation effect was detected. The model suggested that male gamers possess a more positive ATG [*b* = 1.283, *t* = 9.665, *p* < 0.001, 95% CI = (1.022, 1.545)] than male non-gamers, and the positive ATG can predict higher IMPSR toward gamer targets [*b* = 0.268, *t* = 8.684, *p* < 0.001, 95% CI = (0.207, 0.328)], while a significant opposite direct effect was also detected [*b* = −0.715, *t* = −5.824, *p* < 0.001, 95% CI = (−0.956, −0.474)]; see Figure 2). As for the female participants, a full mediation effect was detected. The result suggested that female gamers possess a more positive ATG [*b* = 1.989, *t* = 10.974, *p* < 0.001, 95% CI = (1.633, 2.345)] than female non-gamers, and the positive ATG can predict higher IMPSR toward gamer targets [*b* = 0.190, *t* = 4.437, *p* < 0.001, 95% CI = (0.106, 0.274)]. The direct effect was not significant [*b* = 0.108, *t* = 0.572, *p* = 0.568, 95% CI = (−0.263, −0.478); see Figure 3].

## Discussion

The current study revealed an intergroup bias of non-gamer participants toward gamers in the Chinese marriage market, this result supports our Hypothesis 1, and we also found that female gamer participants showed no preference between gamers and non-gamers when choosing a potential mate while male gamers make higher IMPSR toward gamers than non-gamers additionally. The mediation models indicated that the relatively low IMPSR of non-gamer participants toward gamers is full or partial due to their more negative ATG, this result supports our Hypothesis 2.

These results suggest that “gamer” has become an undesirable label in the context of mate selection for Chinese non-gamers. We argue that this discrimination is due to the stereotypes associated with gaming and various disadvantageous traits which were stereotypically attributed to gamers. In the last 20 years, despite the lack of empirical evidence to support this relationship, many violent crimes, such as shooting accidents, have been blamed on video games (Cunningham et al., 2011, 2016; Quandt et al., 2015). This association has caused an increase in moral panic toward gaming (Markey and Ferguson, 2017), in which gamers are seen as more likely to commit violent crimes. Other studies also indicated that typical gamers were perceived as socially inept a lack of socially competent and lazy (Kowert et al., 2012; Kowert and Oldmeadow, 2012).

In contrast, female gamer participants showed no difference in IMPSR under the two conditions. In other words, whether or not the marriage candidate played video games was not relevant for the selection of a partner. As suggested by previous studies, we argued that the difference in IMPSR between gamer and non-gamer participants under the two conditions was caused by their ATG (Przybylski, 2014; Ferguson et al., 2017). Our findings suggest that non-gamer participants possess a relatively more unfavorable ATG than gamer participants. This result was consistent with previous empirical studies, in which people with concrete game experience exhibited more positive ATG (Przybylski, 2014; Ferguson et al., 2017). Second, among female participants, we found that the effect of participant types on IMPSR toward gamers was fully mediated by ATG, with participants who held more positive ATG producing higher IMPSR toward gamers. This indicates that the differences of IMPSR between gamers and non-gamers were mediated by their attitude toward gaming. Furthermore, we found that male gamers make lower IMPSR toward non-gamers, while the effect of participant types on IMPSR toward gamers was only partial mediated by ATG. We argue that this difference between male and female gamers may also cause by the high gaming engagement of male gamer. For example, people tend to believe that men enjoy playing video games more than women (Colley, 2003), gaming was traditionally perceived as “masculine activity.” For this reason, male gamer may more tend to identify themselves as gamers and see other gamers as in-group member, and finally show “in-group love and out-group hate” (Brewer, 1999, 2001; Schiller et al., 2014) pattern in mate selection.

These results have significant implications. They revealed that, with the development of science and technology, new types of discrimination, that not racial and gender related, deserve our vigilance. It is well known that violent video games, and addiction to games, are widely associated with negative outcomes (Fischer et al., 2010; Kwon et al., 2011; Lemmens et al., 2011; Stetina et al., 2011); however, not all games contain harmful content, and not all gaming is pathological. When gaming is pursued sensibly, it might even provide some benefits. Indeed, some studies have shown that gamers can form new friendships and increase social skills (Longman et al., 2009; Trepte et al., 2012) and enhance cognitive abilities through gaming (Anguera et al., 2013; Boot et al., 2013). Nevertheless, there has been little positive coverage of video games in the mainstream media, which can contribute to biased perceptions of the effects of gaming and in turn, cause unnecessary moral panic. As a relatively young form of entertainment, games have been stigmatized, especially by those without gaming experience (Ferguson, 2008; Przybylski, 2014; Bowman, 2016). Demonizing gaming may lead to antagonism between non-gamers and gamers, and generate more unforeseeable inequities. Finally, it seems that as the number of gamers grows, gamer has gradually become an increasingly large group with great sense of cohesion and identity. We should also be alert to the discrimination of gamers against non-gamers in marriage market, so as to avoid the growing separation between gamers and non-gamers. It is important to recognize that the difference between gamers and non-gamers lies simply in the fact that gamers play video games for entertainment; it should not be a basis for discrimination.

Furthermore, the findings also provided us with insight into preventing this discrimination from becoming a serious social problem. For those who have no concrete gaming experience, becoming familiar with games and learning the effect of gaming on the real world unbiasedly may help them reassess its impact. This may allow them to take an objective view of gaming and, thereby, reduce their misunderstanding toward it.

This study has several limitations. First, we classified potential marriage partners solely based on whether they were gamers. Considering that the attitudes toward different kinds of games may vary, future studies may investigate how game types have an influence on gamers and non-gamers IMPSR. Second, what causes the in-group preference effect of non-gamers when selecting a partner remains unknown; future studies may shed empirical light on the integrated model of this intergroup bias. Third, we did not collect the information about the sexual orientation of participants, the impact of sexual orientation on outcomes remains unknown. Besides, the gamer status was described as “his/her hobby is gaming,” this description may imply a fixation on a single hobby. If participants interpreted the description in this way, this could provide an alternative explanation for the results, as non-gamer participants might have perceived the gamer as only interested in a hobby they did not share. Lastly, we only focused on mate selection attitude in this study, further research may try to examine whether this result can be used to predict actual mate selection behavior and whether such intergroup bias exists in other contexts.

## Data Availability Statement

The raw data supporting the conclusions of this article will be made available by the authors, without undue reservation.

## Ethics Statement

The studies involving human participants were reviewed and approved by the Ethics Committee of Nanjing University. The patients/participants provided their written informed consent to participate in this study.

## Author Contributions

SZ helped supervise the project and wrote the manuscript with support from WZ. SZ and WZ conceived the original idea and carried out the experiment. All authors contributed to the article and approved the submitted version.

## Conflict of Interest

The authors declare that the research was conducted in the absence of any commercial or financial relationships that could be construed as a potential conflict of interest.

## Publisher’s Note

All claims expressed in this article are solely those of the authors and do not necessarily represent those of their affiliated organizations, or those of the publisher, the editors and the reviewers. Any product that may be evaluated in this article, or claim that may be made by its manufacturer, is not guaranteed or endorsed by the publisher.

## Footnotes

^1^ https://www.qualtrics.com/
